# RETRACTION: IκB Kinase Inhibitor VII Modulates Sepsis‐Induced Excessive Inflammation and Cardiac Dysfunction in 5/6 Nephrectomized Mice

**DOI:** 10.1155/mi/9879231

**Published:** 2026-06-28

**Authors:** Mediators of Inflammation

RETRACTION: M. Ding, D. Lian, L. Zhang, T. Jiang, and W. Wang, “IκB Kinase Inhibitor VII Modulates Sepsis‐Induced Excessive Inflammation and Cardiac Dysfunction in 5/6 Nephrectomized Mice”, *Mediators of Inflammation*, vol. 2020 (2020). https://doi.org/10.1155/2020/4251682.

The above article, published online on 10 September 2020 in Wiley Online Library (wileyonlinelibrary.com), has been retracted following the agreement of the Journal’s Chief Editor Anshu Agrawal; and John Wiley & Sons Ltd.

The retraction has been agreed following an investigation undertaken by the publisher. The investigation identified concerns related to the Western Blot data shown in the article as follows:

Figure 4: The bands representing the expression IκBα and Total IκBα in panel (b) appear identical to the bands showing the expression of pSer^32/36^ IκBα and Total IκBα in Figure 4b of a previously published article by different authors [1]. Additional concerns were identified related to the iNOS bands in panel (d), which share similar features with the iNOS panel in Figure 4d of [1].

Figure 8c: The alpha tubulin bands are unexpectedly similar to the bands showing the expression of Total Akt in Figure 7e of [1].

As a result of the investigation, the data and conclusions of this article are considered unreliable. Therefore, the article is retracted.

The authors were informed of this retraction but did not provide a response.
